# Risk for Fomite-Mediated Transmission of SARS-CoV-2 in Child Daycares, Schools, Nursing Homes, and Offices

**DOI:** 10.3201/eid2704.203631

**Published:** 2021-04

**Authors:** Alicia N.M. Kraay, Michael A.L. Hayashi, David M. Berendes, Julia S. Sobolik, Juan S. Leon, Benjamin A. Lopman

**Affiliations:** Emory University, Atlanta, Georgia, USA (A.N.M. Kraay, J.S. Sobolik, J.S. Leon, B.A. Lopman);; University of Michigan, Ann Arbor, Michigan, USA (M.A.L. Hayashi);; Centers for Disease Control and Prevention, Atlanta (D.M. Berendes)

**Keywords:** COVID-19, coronavirus disease, SARS-CoV-2, severe acute respiratory syndrome coronavirus 2, viruses, respiratory infections, zoonoses, fomite, transmission, cleaning, disinfection, daycares, schools, nursing homes, offices, United States

## Abstract

Severe acute respiratory syndrome coronavirus 2 can persist on surfaces, suggesting possible surface-mediated transmission of this pathogen. We found that fomites might be a substantial source of transmission risk, particularly in schools and child daycares. Combining surface cleaning and decontamination with mask wearing can help mitigate this risk.

Severe acute respiratory syndrome coronavirus 2 (SARS-CoV-2), the causative agent of coronavirus disease, can be transmitted through close contact. However, the virus also persists for up to 28 days on surfaces (*1*–*3*), suggesting that surface-mediated (e.g., fomite) transmission might also occur. 

Conventional epidemiologic studies cannot distinguish between competing transmission pathways (e.g., droplet or fomite) when they act simultaneously. Therefore, we used a transmission model to explore the potential for fomite transmission without other pathways. We adapted a published fomite transmission model (*4*) for SARS-CoV-2 (Appendix Figure 1). In our model, persons are classified as susceptible, infectious, or recovered. We explicitly tracked contamination on hands, which is independent of whether or not a person is currently infected. Infectious persons shed pathogens onto fomites or hands, but only a fraction of surfaces (λ) are accessible for contamination. Hands might become contaminated from viral excretion or from touching virus-contaminated fomites. Susceptible persons might become infected through touching their face and mouth with contaminated hands (Appendix).

By using this model, we explore how fomite transmission varies by location (comparing child daycares, schools, offices, and nursing homes), disinfection strategy, and surface type. Although precise values likely vary on a case-by-case basis, child daycares are assumed to have higher frequency of fomite touching (*ρ_T_*) and the fraction of surfaces susceptible to contamination (λ) than offices, whereas schools are likely intermediate for both factors (*4*). Nursing homes are assumed to have similar amounts of surfaces susceptible to contamination to offices, but higher fomite touching rates.

We considered the following surface cleaning and disinfection frequencies: every 8 hours (1×/workday), every 4 hours (2×/workday), and hourly. We also considered handwashing interventions, but they had minimal impact in our model and were not included in our main results (Appendix). Because SARS-CoV-2 persistence varies by surface, we compared transmission for stainless steel, plastic, and cloth. As a sensitivity analysis, we also varied viral shedding rates in our analysis for 2 reasons: initial data are uncertain because of small sample sizes (*5*), and shedding rates are likely to vary on the basis of mask-wearing practices (*6**,**7*; Appendix). In our model, situations in which the basic reproduction number (R_0_) for the fomite route exceeds 1 could sustain ongoing transmission in a given setting, whereas transmission could be interrupted when R_0_ falls below 1. We explored what interventions could interrupt fomite transmission.

Our estimates suggest that fomite transmission could sustain SARS-CoV-2 transmission in many settings. The fomite R_0_ ranged from 10 in low-risk venues (offices) to ≈25 in high-risk settings such as child daycares. SARS-CoV-2 transmission risk is generally higher than influenza and rhinovirus (Appendix Figure 6).

We found that hourly cleaning and disinfection alone could interrupt fomite transmission in some office settings, particularly combined with reduced shedding, but would be inadequate in child daycares and schools (Figure; Appendix Figure 3). If shedding is reduced through mask wearing, transmission from surfaces became unlikely, even with infrequent surface decontamination. Decay rates were similarly low for plastic and stainless steel (Appendix Table 2), leading to substantial transmission potential (Figure). Decay rates on cloth were high and were unlikely to sustain transmission. Therefore, cleaning and disinfection frequencies could vary by surface, with hourly interventions being helpful for frequently touched nonporous surfaces and with porous surfaces (such as plush toys) being cleaned and sanitized less frequently. In child daycares, intervening directly after high-risk shedding events (e.g., a feverish person coughs directly on a surface) in addition to intervening at standard intervals (such as hourly) would be beneficial.

**Figure:**
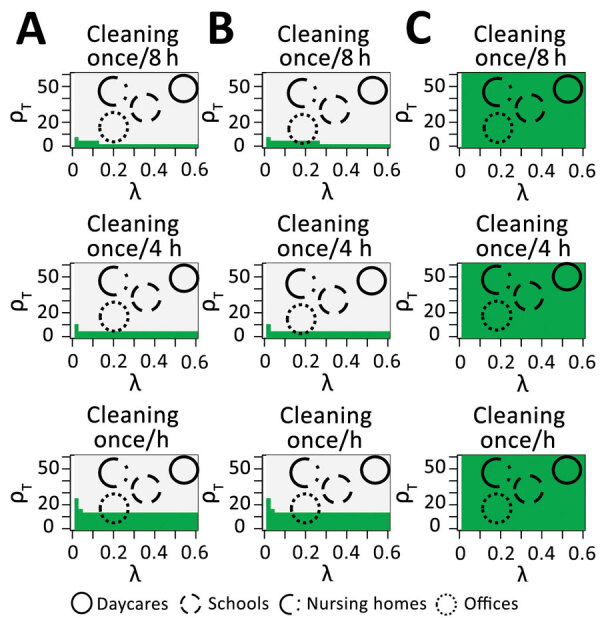
Reductions in the basic reproduction number for the fomite pathway for severe acute respiratory syndrome coronavirus 2 on stainless steel (A), plastic (B), and cloth surfaces (C), by setting (defined by hourly fomite touching rates [*ρ_T_*and proportion of accessible surfaces [λ). For areas in green, the projected reproduction number from fomite transmission is <1. For comparison, cleaning every 2 hours was considered as a sensitivity analysis.

Because of our emphasis on the basic reproduction number rather than simulating infection dynamics, these results describe transmission potential if outbreaks begin with a single case as opposed to many cases being introduced simultaneously, which could occur when transmission is high. Thus, these results apply when SARS-CoV-2 incidence is low, which might be achievable in individual locations even if community incidence is high. Near the epidemic peak, more detailed simulations are needed because environmental contamination might exceed the linear range of the dose-response curve (*8*), which could lead to an overestimate of the risk for fomite transmission. Because our objective was to assess the potential impact of fomite transmission alone, we did not account for direct transmission through direct droplet spray, aerosols, or hand-to-hand contact, all of which are likely major contributors to transmission in many settings (*9*). Our model suggests fomites can also transmit virus, which is important for indirect exposures. For simplicity, we assume that fomite transmission is similar for symptomatic and asymptomatic infections (Appendix). We also assume that the dose-response curve for fomite transmission is the same as other transmission routes, which might lead to an overestimate of fomite transmission if pathogens from surfaces are less efficiently absorbed into the lungs from hands when they are not aerosolized.

In summary, fomite transmission might be an important source of risk for SARS-CoV-2. However, both mask wearing and frequent cleaning and disinfection can reduce this risk.

Appendix Additional information about risk for fomite-mediated transmission of SARS-CoV-2 in child daycares, schools, nursing homes, and offices.
