# Supplementary material for: Risk for Fomite-Mediated Transmission of SARS-CoV-2 in Child Daycares, Schools, Nursing Homes, and Offices
Source: Emerg Infect Dis. 2021 Apr;27(4):1229–31. doi: 10.3201/eid2704.203631 (PMC8007300; doi:10.3201/eid2704.203631)
Supplement: Appendix — Additional information about risk for fomite-mediated transmission of SARS-CoV-2 in child daycares, schools, nursing homes, and offices. [file 20-3631-Techapp-s1.pdf]

Article DOI: https://doi.org/10.3201/eid2704.203631

Risk for Fomite-Mediated Transmission of SARS-CoV-2 in Child Daycares, Schools, Nursing Homes, and Offices

Appendix

Model Details

This model has previously been described in detail, but in brief, the population is classified as susceptible (S), infectious (I), or recovered (R). Infectious individuals can shed pathogens directly onto fomites (F) or onto hands (H compartments, either H_S, H_I, or H_R). Hands have the same infection status as individuals (i.e., all susceptible individuals, S, have susceptible hands H_S). Hands may become contaminated through direct excretion (self-inoculation) by an infectious individual or through touching contaminated fomites. Hand contamination is not dependent on an individual's infection status, thus, a recovered individual (H_R) could have contaminated hands. Susceptible individuals can become infected through touching their face and/or mouth with contaminated hands (self-inoculation) based on the linear dose-response function, P. Pathogens on hands and on fomites are inactivated based on the persistence properties of the pathogen. In addition to natural inactivation, pathogens may be removed from hands or surfaces through cleaning interventions, which is determined by both the frequency and efficacy of the cleaning. The parameter values considered in this model and their definitions are shown in Appendix Table 1. The model equations and the corresponding R_0 equations are shown below. A model diagram is shown in Appendix Figure 1.

In our model, we assume a cleaning and decontamination efficacy of 100%, consistent with initial data on the efficacy of standard cleaning agents (*1*). However, if cleaning without disinfection is used (i.e., removing only organic matter on surfaces without using disinfection agents, such as bleach), higher frequencies of cleaning would be unlikely to reduce risk unless pathogen shedding on surfaces can also be substantially reduced.

$$\frac{dS}{dt} = -\rho P\left(\frac{\chi H_S}{S}\right)S$$

$$\frac{dI}{dt} = \rho P\left(\frac{\chi H_S}{S}\right)S - \gamma I$$

$$\frac{dR}{dt} = \gamma I$$

$$\frac{dF}{dt} = \alpha_F I - (\rho_{FH} N + \mu_F + \theta_F)F + \rho_{HF}(H_S + H_I + H_R)$$

$$\frac{dH_S}{dt} = \rho_{FH} SF - (\mu_H + \rho_{HF} + \chi\rho + \theta_H)H_S - (1-\chi)\rho P\left(\frac{\chi H_S}{S}\right)H_S$$

$$\frac{dH_I}{dt} = \rho_{FH} IF - (\mu_H + \rho_{HF} + \chi\rho + \theta_H)H_I + \alpha_H I + (1-\chi)\rho\, P\left(\frac{\chi H_S}{S}\right)H_S - \gamma H_I$$

$$\frac{dH_R}{dt} = \rho_{FH} RF - (\mu_H + \rho_{HF} + \chi\rho + \theta_H)H_R + \gamma H_I$$

$$R_0 = \frac{1}{\gamma}\left[\frac{\chi\rho\pi}{\mu_H + \rho_{HF} + \chi\rho + \theta_H}\right]\left[\left(\frac{\frac{\rho_{FH} N}{\rho_{FH} N + \mu_F + \theta_F}}{1 - \frac{\rho_{HF}\rho_{FH} N}{(\mu_H + \rho_{HF} + \chi\rho + \theta_H)(\rho_{FH} N + \mu_F + \theta_F)}}\right)\left(\alpha_F + \alpha_H\left[\frac{\rho_{HF}}{\mu_H + \rho_{HF} + \chi\rho + \theta_H}\right]\right) + \alpha_H\right]$$

Deriving model parameters for SARS-CoV-2 and uncertainty

For unknown persistence and transfer efficiency parameters, we used influenza values because SARS-CoV-2, like influenza, is an enveloped virus, which tend to have lower persistence than non-enveloped viruses on surfaces (*34*). For uncertain infectivity parameters, we used values from other coronaviruses (if available) or rhinovirus because of the similarity in symptoms likely to drive transmission.

Infectivity parameters—shedding rate and dose response curves

To provide the most accurate infectivity data, we used shedding rates that were in terms of $TCID_{50}$ units, as this excludes potential bias from measuring genetic material from inactive virus. $TCID_{50}$ units describe the concentration of virus that would be expected to infect 50% of challenged cells, whereas PFU units describe the number (count) of plaques likely to be formed

in a cell culture containing a given concentration of infectious virus. Based on this definition, when $TCID_{50} = 1$, the expected number of observed plaques is 0. Therefore, for parameters in PFU units, we converted between the two scales as follows, applying the Poisson distribution, where the expected number of plaques observed is dependent on μ, the PFU/mL (*35*,*36*):

$$P(Count\ of\ plaques = y) \sim Pois(\mu = PFU) = \frac{e^{-\mu}\mu^{y}}{y!}$$

$$P(Count\ of\ plaques = 0) = \frac{e^{-\mu}\mu^{0}}{0!}$$

$$0.5\ TCID_{50}/mL = e^{-PFU}$$

Thus,

$$TCID_{50} = -\ln(0.5)\ PFU$$

Decay rates on fomites

For SARS-CoV-2, decay rates on different surfaces were calculated based on the slope of the decay curves first 24 hours for cloth (highest decay rates), plastic (lowest decay rates) and stainless steel (medium decay rates). For cloth, the decay rates were calculated based on the first 30 minutes of decay because most samples became undetectable after the first 30 minutes of observation and were then multiplied by 2 to get the corresponding hourly decay rates. Because experimental data was presented on the log10 scale, the decay rates from the different experimental studies were initially calculated on the log10 scale. To convert these numbers to the exponential scale, the decay rate on the log10 scale was multiplied by ln(10).

Decay rates on hands

For SARS-CoV-2, there were no reliable pathogen-specific data for the decay rate of pathogen on hands. One study used skin samples from corpses to estimate this quantity and calculated decay rates for both influenza and SARS-CoV-2. In this study, decay rates on hands were substantially lower for SARS-CoV-2 compared with influenza, with SARS-CoV-2 surviving for about eight times as long as influenza (*22*). However, the decay rates from influenza obtained using this method differed substantially from prior decay rate estimates for influenza (*14*,*21*), casting doubt on this model system for estimating the absolute decay rates. Moreover, this study used a method that pipetted pathogen directly onto hands rather than first

transferring pathogen from an inanimate surface to hands. Given that decay rates on hands vary based on the surface from which they are transferred (even after accounting for transfer efficacy) (*21*), these data were inappropriate for use in our fomite model. For this reason, we relied on decay rates previously estimated for influenza and considered a lower hand decay rate as a sensitivity analysis, where the decay rate on hands for SARS-CoV-2 was 11.02/hour, ≈1/8 the decay rate previously estimated for influenza.

Transfer efficacy

For SARS-CoV-2, there were no data available on transfer efficacy, so we relied on the transfer efficacy parameters previously estimated for influenza.

Handwashing

We initially considered a handwashing intervention that removed all pathogens on hands at regular intervals. However, SARS-CoV-2 is predicted to have very low persistence on hands (Appendix Table 1). For this reason, even hourly handwashing with 100% effectiveness had almost no impact on transmission risk. To illustrate, we reran Figure (https://wwwnc.cdc.gov/EID/article/27/4/20-3631-F1.htm) assuming hourly handwashing and the results were nearly identical to the version in the main text with no handwashing (Appendix Figure 2).

Shedding Sensitivity Analysis

Baseline hand persistence

As a sensitivity analysis, we considered how the basic reproduction number would change if the hourly shedding rate were slightly different, using stainless steel as the surface for comparison. Preliminary data suggest a shedding rate of 1800 pathogens/person/hr (1.8E3) (*6*), which is the estimate used in the main text. We considered 2E3 as a potential upper bound on shedding for SARS-CoV-2 (Appendix Figure 3). Widespread use of masks might also lower the overall shedding rate. To approximate the impact of mask wearing, we reduced both shedding (α) and the rate of self-inoculation (ρ) by 75% ($\alpha = 450, \rho = 3.95$), consistent with estimates of viral reduction in droplets during speech with mask wearing (*13*).

Shedding might be lower or higher than expected for several reasons. First, initial studies were limited in sample size, which may have reduced the precision and accuracy of early

estimates. In addition, overall shedding might vary by level of symptoms. While viral load has been found to be similar in secretions of symptomatic and asymptomatic cases, they may not have similar contributions to overall transmission if shedding is influenced strongly by symptoms. For example, the number of droplets expelled during coughing, a common symptom of SARS-CoV-2, is much higher than during speech (*37*). On the other hand, symptomatic individuals may be more likely to take precautions to limit viral spread, making asymptomatic transmission relatively more important. With mask wearing, transmission from surfaces was unlikely, even when surface decontamination was set to its minimum value (once every 8 hours).

Higher hand persistence

We repeated the same sensitivity analysis varying shedding while also allowing persistence to be higher, based on data from (*22*). When hand persistence was higher, transmission from surfaces could not be controlled unless both mask wearing and frequent surface disinfection were used (Appendix Figure 4). Even with hourly handwashing and mask wearing, transmission from surfaces would only be controlled in office settings.

Alternative cleaning frequencies

For comparison, we reran Figure 2 considering cleaning frequencies every 2 hours (Appendix Figure 5). The impacts were intermediate between cleaning every 4 hours and hourly cleaning.

References

1. Chin AWH, Chu JTS, Perera MRA, Hui KPY, Yen HL, Chan MCW, et al. Stability of SARS-CoV-2 in different environmental conditions. Lancet Microbe. 2020;1:e10. PubMed https://doi.org/10.1016/S2666-5247(20)30003-3

2. Hayden FG, Fritz R, Lobo MC, Alvord W, Strober W, Straus SE. Local and systemic cytokine responses during experimental human influenza A virus infection. Relation to symptom formation and host defense. J Clin Invest. 1998;101:643–9. PubMed https://doi.org/10.1172/JCI1355

3. Suess T, Buchholz U, Dupke S, Grunow R, an der Heiden M, Heider A, et al.; Robert Koch Institute Shedding Investigation Group. Shedding and transmission of novel influenza virus A/H1N1 infection in households—Germany, 2009. Am J Epidemiol. 2010;171:1157–64. PubMed https://doi.org/10.1093/aje/kwq071

4. Suess T, Remschmidt C, Schink SB, Schweiger B, Heider A, Milde J, et al. Comparison of shedding characteristics of seasonal influenza virus (sub)types and influenza A(H1N1)pdm09; Germany, 2007-2011. PLoS One. 2012;7:e51653. PubMed https://doi.org/10.1371/journal.pone.0051653

5. Peltola V, Waris M, Kainulainen L, Kero J, Ruuskanen O. Virus shedding after human rhinovirus infection in children, adults and patients with hypogammaglobulinaemia. Clin Microbiol Infect. 2013;19:E322–7. PubMed https://doi.org/10.1111/1469-0691.12193

6. Bullard J, Dust K, Funk D, Strong JE, Alexander D, Garnett L, et al. Predicting infectious SARS-CoV-2 from diagnostic samples. Clin Infect Dis. 2020 May 22 [Epub ahead of print]. PubMed

7. Byrne AW, McEvoy D, Collins AB, Hunt K, Casey M, Barber A, et al. Inferred duration of infectious period of SARS-CoV-2: rapid scoping review and analysis of available evidence for asymptomatic and symptomatic COVID-19 cases. BMJ Open. 2020;10:e039856. PubMed https://doi.org/10.1136/bmjopen-2020-039856

8. Lee N, Chan PK, Hui DS, Rainer TH, Wong E, Choi KW, et al. Viral loads and duration of viral shedding in adult patients hospitalized with influenza. J Infect Dis. 2009;200:492–500. PubMed https://doi.org/10.1086/600383

9. Hall CB, Douglas RG Jr, Geiman JM, Meagher MP. Viral shedding patterns of children with influenza B infection. J Infect Dis. 1979;140:610–3. PubMed https://doi.org/10.1093/infdis/140.4.610

10. Lau LL, Cowling BJ, Fang VJ, Chan KH, Lau EH, Lipsitch M, et al. Viral shedding and clinical illness in naturally acquired influenza virus infections. J Infect Dis. 2010;201:1509–16. PubMed https://doi.org/10.1086/652241

11. Hendley JO, Gwaltney JMJ Jr. Viral titers in nasal lining fluid compared to viral titers in nasal washes during experimental rhinovirus infection. J Clin Virol. 2004;30:326–8. PubMed https://doi.org/10.1016/j.jcv.2004.02.011

12. Douglas RGJ Jr, Cate TR, Gerone PJ, Couch RB. Quantitative rhinovirus shedding patterns in volunteers. Am Rev Respir Dis. 1966;94:159–67. PubMed

13. Fischer EP, Fischer MC, Grass D, Henrion I, Warren WS, Westman E. Low-cost measurement of face mask efficacy for filtering expelled droplets during speech. Sci Adv. 2020;6:eabd3083. PubMed https://doi.org/10.1126/sciadv.abd3083

14. Bean B, Moore BM, Sterner B, Peterson LR, Gerding DN, Balfour HH Jr. Survival of influenza viruses on environmental surfaces. J Infect Dis. 1982;146:47–51. PubMed https://doi.org/10.1093/infdis/146.1.47

15. Noyce JO, Michels H, Keevil CW. Inactivation of influenza A virus on copper versus stainless steel surfaces. Appl Environ Microbiol. 2007;73:2748–50. PubMed https://doi.org/10.1128/AEM.01139-06

16. Weber TP, Stilianakis NI. Inactivation of influenza A viruses in the environment and modes of transmission: a critical review. J Infect. 2008;57:361–73. PubMed https://doi.org/10.1016/j.jinf.2008.08.013

17. Boone SA, Gerba CP. Significance of fomites in the spread of respiratory and enteric viral disease. Appl Environ Microbiol. 2007;73:1687–96. PubMed https://doi.org/10.1128/AEM.02051-06

18. Hendley JO, Wenzel RP, Gwaltney JM Jr. Transmission of rhinovirus colds by self-inoculation. N Engl J Med. 1973;288:1361–4. PubMed https://doi.org/10.1056/NEJM197306282882601

19. van Doremalen N, Bushmaker T, Morris DH, Holbrook MG, Gamble A, Williamson BN, et al. Aerosol and surface stability of SARS-CoV-2 compared with SARS-CoV-1. N Engl J Med. 2020;382:1564–7. PubMed https://doi.org/10.1056/NEJMc2004973

20. Nicas M, Jones RM. Relative contributions of four exposure pathways to influenza infection risk. Risk Anal. 2009;29:1292–303. PubMed https://doi.org/10.1111/j.1539-6924.2009.01253.x

21. Ansari SA, Springthorpe VS, Sattar SA, Rivard S, Rahman M. Potential role of hands in the spread of respiratory viral infections: studies with human parainfluenza virus 3 and rhinovirus 14. J Clin Microbiol. 1991;29:2115–9. PubMed https://doi.org/10.1128/JCM.29.10.2115-2119.1991

22. Hirose R, Ikegaya H, Naito Y, Watanabe N, Yoshida T, Bandou R, et al. Survival of severe acute respiratory syndrome coronavirus 2 (SARS-CoV-2) and influenza virus on human skin: importance of hand hygiene in coronavirus disease 2019 (COVID-19). Clin Infect Dis. 2020 Oct 3 [Epub ahead of print]. https://doi.org/10.1093/cid/ciaa1517

23. Greene C, Vadlamudi G, Eisenberg M, Foxman B, Koopman J, Xi C. Fomite-fingerpad transfer efficiency (pick-up and deposit) of *Acinetobacter baumannii*-with and without a latex glove. Am J Infect Control. 2015;43:928–34. PubMed https://doi.org/10.1016/j.ajic.2015.05.008

24. Rusin P, Maxwell S, Gerba C. Comparative surface-to-hand and fingertip-to-mouth transfer efficiency of gram-positive bacteria, gram-negative bacteria, and phage. J Appl Microbiol. 2002;93:585–92. PubMed https://doi.org/10.1046/j.1365-2672.2002.01734.x

25. Lopez GU. Transfer of microorganisms from fomites to hands and risk assessment of contaminated and disinfected surfaces. Tucson (Arizona): The University of Arizona; 2013 [cited 2021 Jan 27]. https://repository.arizona.edu/handle/10150/272839

26. Pancic F, Carpentier DC, Came PE. Role of infectious secretions in the transmission of rhinovirus. J Clin Microbiol. 1980;12:567–71. PubMed https://doi.org/10.1128/JCM.12.4.567-571.1980

27. Reed SE. An investigation of the possible transmission of Rhinovirus colds through indirect contact. J Hyg (Lond). 1975;75:249–58. PubMed https://doi.org/10.1017/S0022172400047288

28. Kraay ANM, Hayashi MAL, Hernandez-Ceron N, Spicknall IH, Eisenberg MC, Meza R, et al. Fomite-mediated transmission as a sufficient pathway: a comparative analysis across three viral pathogens. BMC Infect Dis. 2018;18:540. PubMed https://doi.org/10.1186/s12879-018-3425-x

29. Quantitative Microbial Risk Assessment Wiki. SARS dose response experiments. 2013 [cited 2021 Jan 27]. http://qmrawiki.org/experiments/sars.

30. Dediego ML, Pewe L, Alvarez E, Rejas MT, Perlman S, Enjuanes L. Pathogenicity of severe acute respiratory coronavirus deletion mutants in hACE-2 transgenic mice. Virology. 2008;376:379–89. PubMed https://doi.org/10.1016/j.virol.2008.03.005

31. De Albuquerque N, Baig E, Ma X, Zhang J, He W, Rowe A, et al. Murine hepatitis virus strain 1 produces a clinically relevant model of severe acute respiratory syndrome in A/J mice. J Virol. 2006;80:10382–94. PubMed https://doi.org/10.1128/JVI.00747-06

32. Elder NC, Sawyer W, Pallerla H, Khaja S, Blacker M. Hand hygiene and face touching in family medicine offices: a Cincinnati Area Research and Improvement Group (CARInG) network study. J Am Board Fam Med. 2014;27:339–46. PubMed https://doi.org/10.3122/jabfm.2014.03.130242

33. Nicas M, Best D. A study quantifying the hand-to-face contact rate and its potential application to predicting respiratory tract infection. J Occup Environ Hyg. 2008;5:347–52. PubMed https://doi.org/10.1080/15459620802003896

34. Modrow S, Falke D, Truyen U, Schätzl H. Viruses: definition, structure, classification. In: Molecular Virology. Berlin: Springer; 2013. p. 17–30.

35. Carter J, Saunders V. Virology: principles and applications. 2nd edition. Chichester (United Kingdom): John Wiley & Sons; 2013.

36. American Type Culture Collection. Converting $TCID_{50}$ to plaque forming units (PFU). Is it possible to determine from the $TCID_{50}$ how many plaque forming units to expect? 2012 Jul 25 [cited 2021 Jan 27]. https://www.atcc.org/en/Global/FAQs/4/8/Converting_TCID50_to_plaque_forming_units_PFU-124.aspx

37. Jayaweera M, Perera H, Gunawardana B, Manatunge J. Transmission of COVID-19 virus by droplets and aerosols: A critical review on the unresolved dichotomy. Environ Res. 2020;188:109819. PubMed https://doi.org/10.1016/j.envres.2020.109819

Appendix Table 1. List of pathogen-specific parameters values and references*

Parameter type	Influenza	Rhinovirus	SARS-CoV-2
Pathogen-specific parameters			
$1/\gamma$: Infectious period (days)	6 (*2–4*)	10.4 (*5*)	8 (*6,7*)
α: Shedding rate (pathogen hours^{-1} people^{-1})	1×10^4 *(8–10)*	1×10^3 *(11,12)*	1.8×10^3 *(6,13)* ($450, 2 \times 10^3$)
μ_F: Inactivation rate in fomites (hours^{-1})	0.121 (*14–16*)	1.44 (*17,18*)	0.148 (*1,19*) (0.131, 9.21)
μ_H: Inactivation rate in hands (hours^{-1})	88.2 (*14,16,20*)	0.767 (*21*)	88.2 (*14,16,20,22*)
τ_{FH}: *Transfer efficacy (F to H) (proportion)*	0.1 (*14,20,23,24*)	0.2 (*21,25–27*)	**0.1** (*14,20,23,24*)
τ_{HF}: *Transfer efficacy (H to F) (proportion)*	0.025 (*14,20,23,24*)	0.2 (*21,25–27*)	**0.025** (*14,20,23,24*)
ϕ_H: *Pathogens excreted to hands (proportion)*	0.15	0.15	0.15
ϕ_F: *Pathogens excreted to fomites (proportion)*	$1 - \phi_H$	$1 - \phi_H$	$1 - \phi_H$
π: Infectivity parameter in contact with x pathogens (unitless, $P'(0)$)†	6.93×10^{-5} *(28)*	2.46×10^{-3} *(28)*	3.55×10^{-3} *(29–31)*
α_H: rate pathogens are added to hands (pathogen hours^{-1} people^{-1})	$\alpha\phi_H$	$\alpha\phi_H$	$\alpha\phi_H$
Venue-specific parameters			
λ: Accessible surfaces (proportion)	(0, 0.6)	(0, 0.6)	(0, 0.6)
κ: fingertip to surface ratio per individual (1/people)‡	$\frac{6 \times 10^{-6}}{\lambda}$	$\frac{6 \times 10^{-6}}{\lambda}$	$\frac{6 \times 10^{-6}}{\lambda}$
ρ_T: rate of fomite touching (hours^{-1})	(0, 60)	(0, 60)	(0, 60)
ρ_{FH}: *rate of fomite pick up from fomites to hand (1/(hours x people))*	$\rho_T\tau_{FH}\kappa$	$\rho_T\tau_{FH}\kappa$	$\rho_T\tau_{FH}\kappa$
ρ_{HF}: *rate of fomite pick up from hands to fomites (1/(hours x people))*	$\rho_T\tau_{HF}$	$\rho_T\tau_{HF}$	$\rho_T\tau_{HF}$
α_F: rate pathogens are added to fomites (pathogen hours^{-1} people)	$\alpha\phi_F\lambda$	$\alpha\phi_F\lambda$	$\alpha\phi_F\lambda$
Cleaning parameters			
Θ_F: Rate of fomite cleaning (hours^{-1})	(1/8, 1)	(1/8, 1)	(1/8, 1)
Θ_H: Rate of hand cleaning (hours^{-1})	(1/8, 1)	(1/8, 1)	(1/8, 1)
q_F: *Fomite cleaning efficacy (proportion)*	1	1	1
q_H: *Hand cleaning efficacy (proportion)*	1	1	1
θ_F: Effective fomite cleaning rate (hours^{-1})	$q_F\Theta_F$	$q_F\Theta_F$	$q_F\Theta_F$
θ_H: Effective hand cleaning rate (hours^{-1})	$q_H\Theta_H$	$q_H\Theta_H$	$q_H\Theta_H$
Fixed parameters (across pathogens and venues)			
ρ: Self-inoculation (hours^{-1})	15.8 (*32,33*)	15.8 (*32,33*)	15.8 (3.95, 15.8) (*13,32,33*)
χ: Proportion of pathogens absorbed when self-inoculation occurs (proportion)	1	1	1

*Parameter values for rhinovirus and influenza are shown for comparison. A range is also included for parameters that were used to perform a sensitivity analysis (frequency of cleaning, infectious period, shedding rate, and persistence on surfaces). Derived parameters are shown as a function of the parameters used to derive them. Decay rates on fomites for influenza and rhinovirus are for stainless steel. For SARS-CoV-2, the decay rates shown in parentheses are the range of observed for plastic (lowest decay rates), stainless steel, and cloth (highest decay rates). For SARS-CoV-2, parameters that were extrapolated based on data from other pathogens are shown in bold.
†Parameter fixed based on linearization of the dose-response curve, P.
‡Parameter fixed based on relative finger to body size

Appendix Table 2. Decay rates for each surface*

Surface	Doremalen et al. (*19*)	Chin et al. (*1*)	Average
Stainless steel (ln/hr)	0.170	0.092	0.131
Plastic (ln/hr)	0.108 (3.64, 2.52)	0.184	0.146
Cloth (ln/hr)	NA	9.21	9.21

*NA, not available.

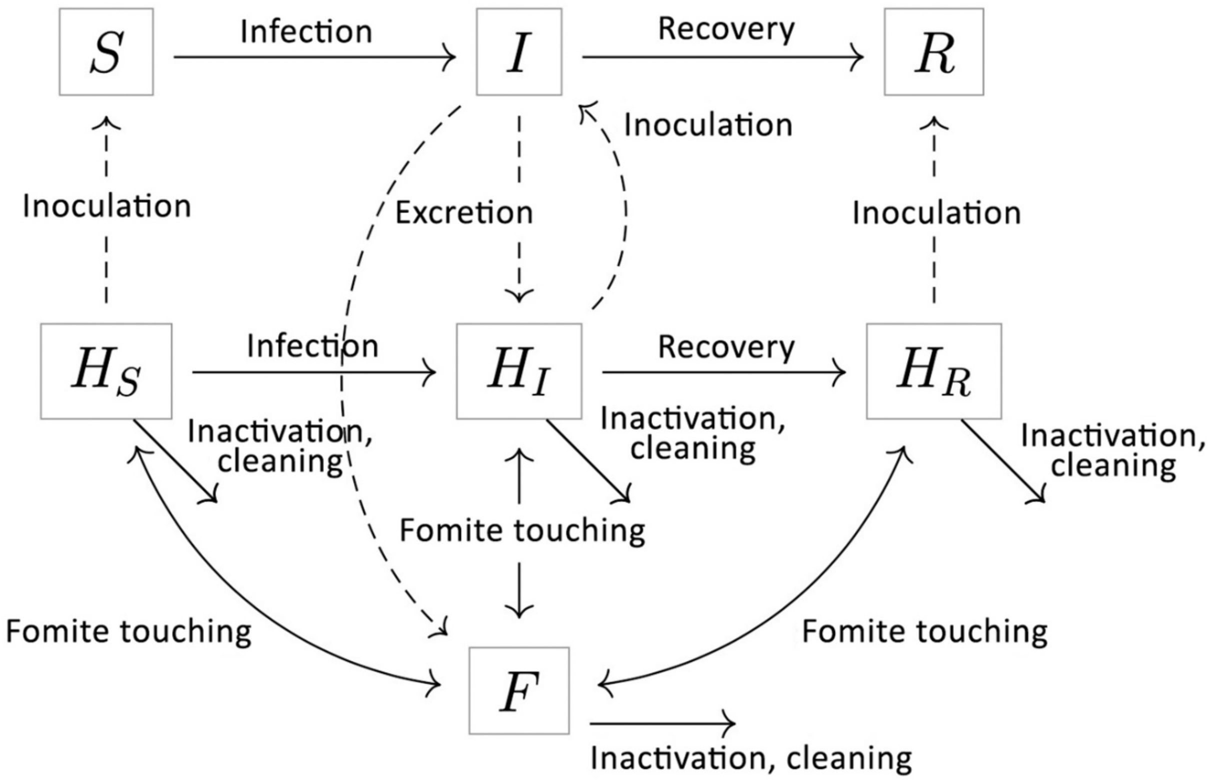

Appendix Figure 1. Model tracks individuals (in compartments *S*, *I* or *R*) and pathogens on fomites (*F*) and hands (hands of susceptible individuals, H_S; hands of infected individuals, H_I; hands of recovered individuals; H_R). The six events (inoculation, fomite touching, excretion, pathogen inactivation, cleaning, and recovery) are represented by arrows in the direction of the corresponding flow.

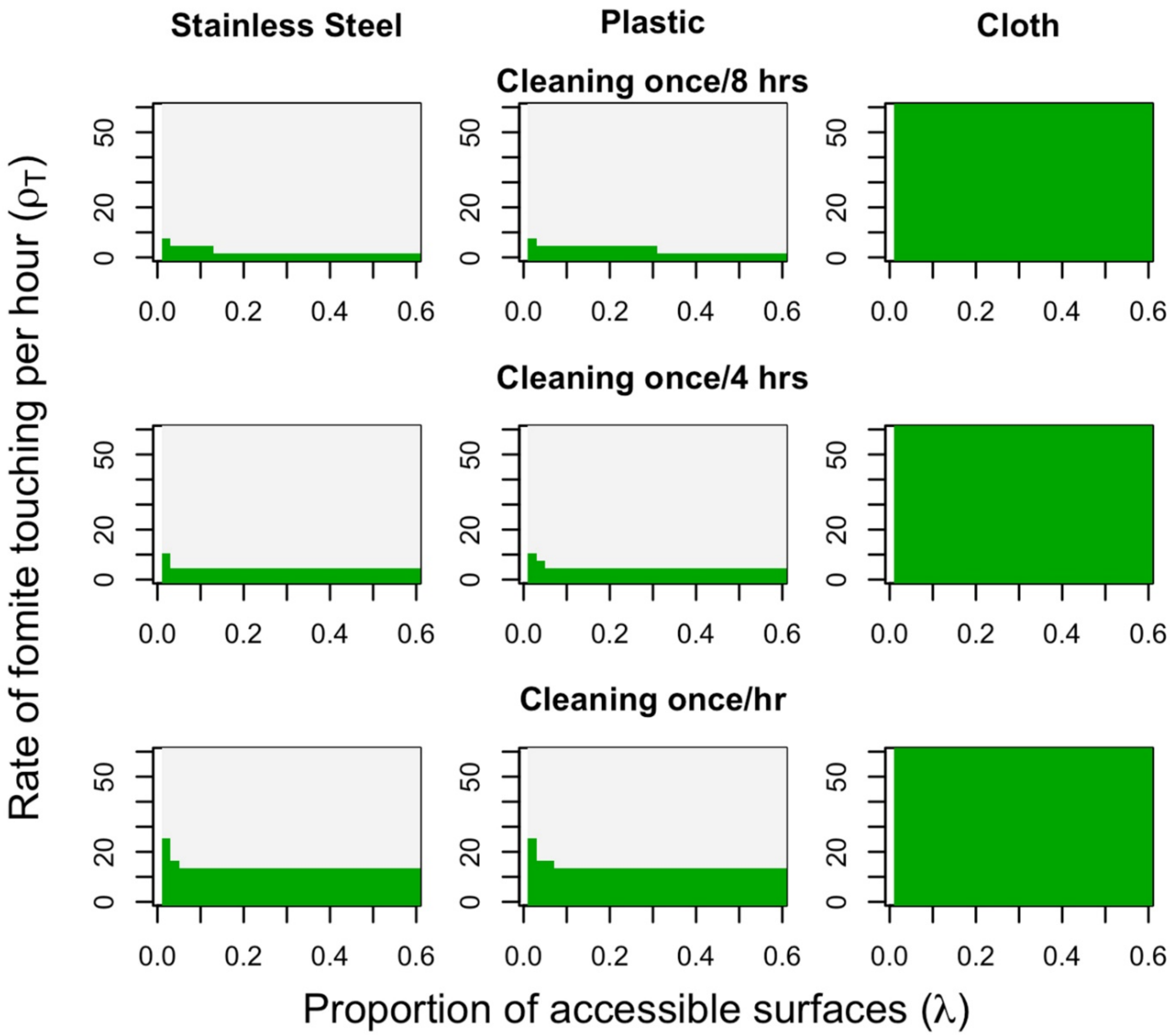

Appendix Figure 2. Reductions in the basic reproduction number by cleaning strategy and surface with hourly handwashing included. For areas in green, the projected reproduction number from fomite transmission is below 1.

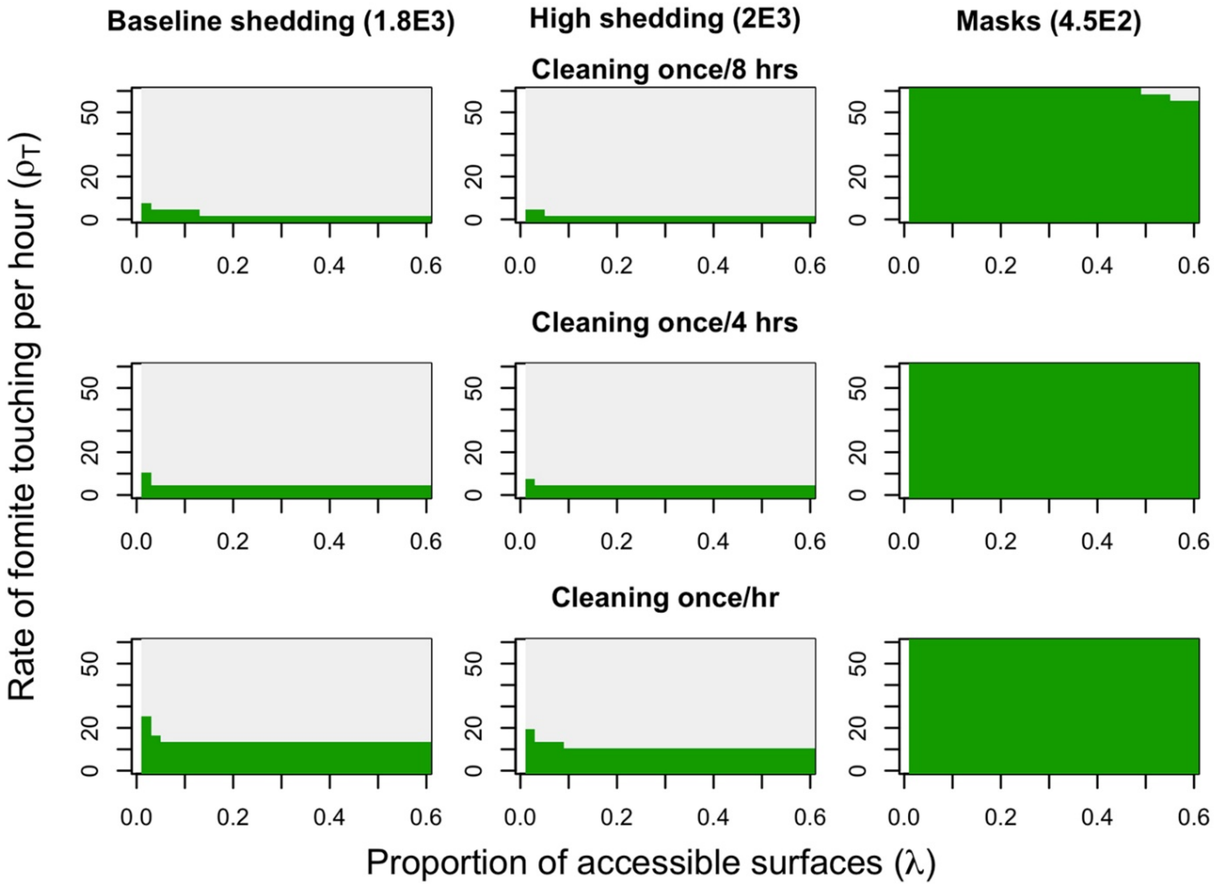

Appendix Figure 3. Reductions in the basic reproduction number by cleaning strategy for different shedding rates (baseline, upper bound of shedding, and mask wearing). For areas in green, the projected reproduction number from fomite transmission is below 1.

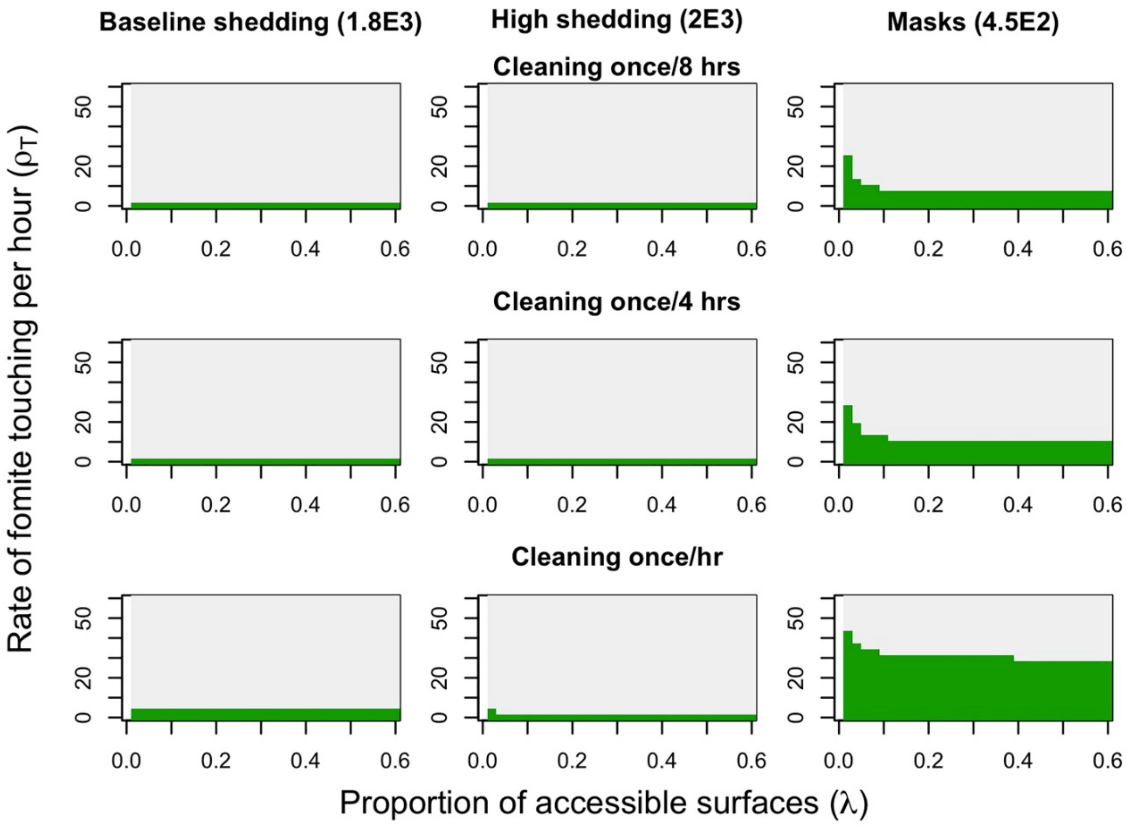

Appendix Figure 4. Reductions in the basic reproduction number by cleaning strategy for different shedding rates (baseline, upper bound of shedding, and mask wearing) for stainless steel surfaces assuming higher hand persistence. For areas in green, the projected reproduction number from fomite transmission is below 1.

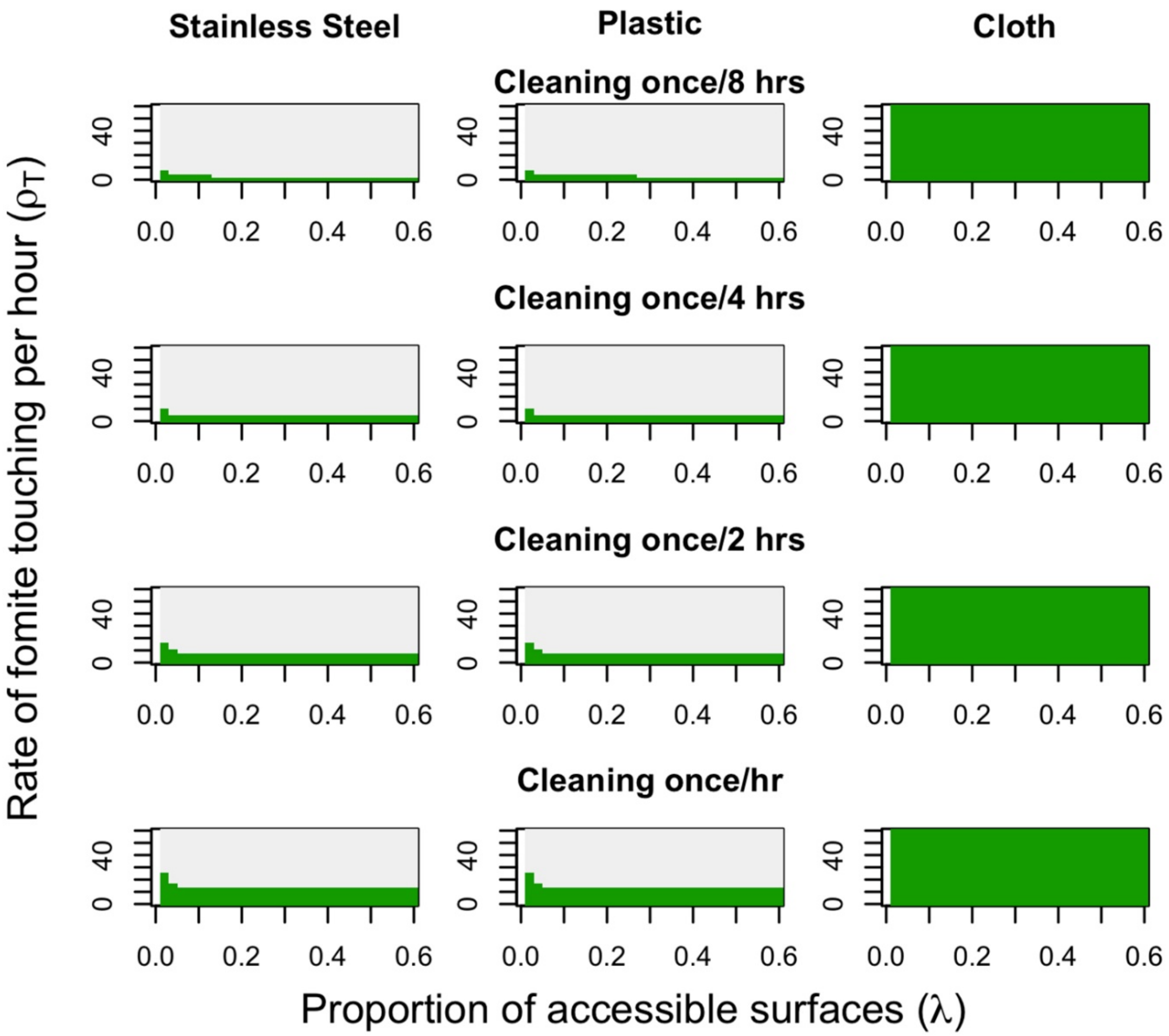

Appendix Figure 5. Reductions in the basic reproduction number for different surfaces. For areas in green, the projected reproduction number from fomite transmission is below 1.

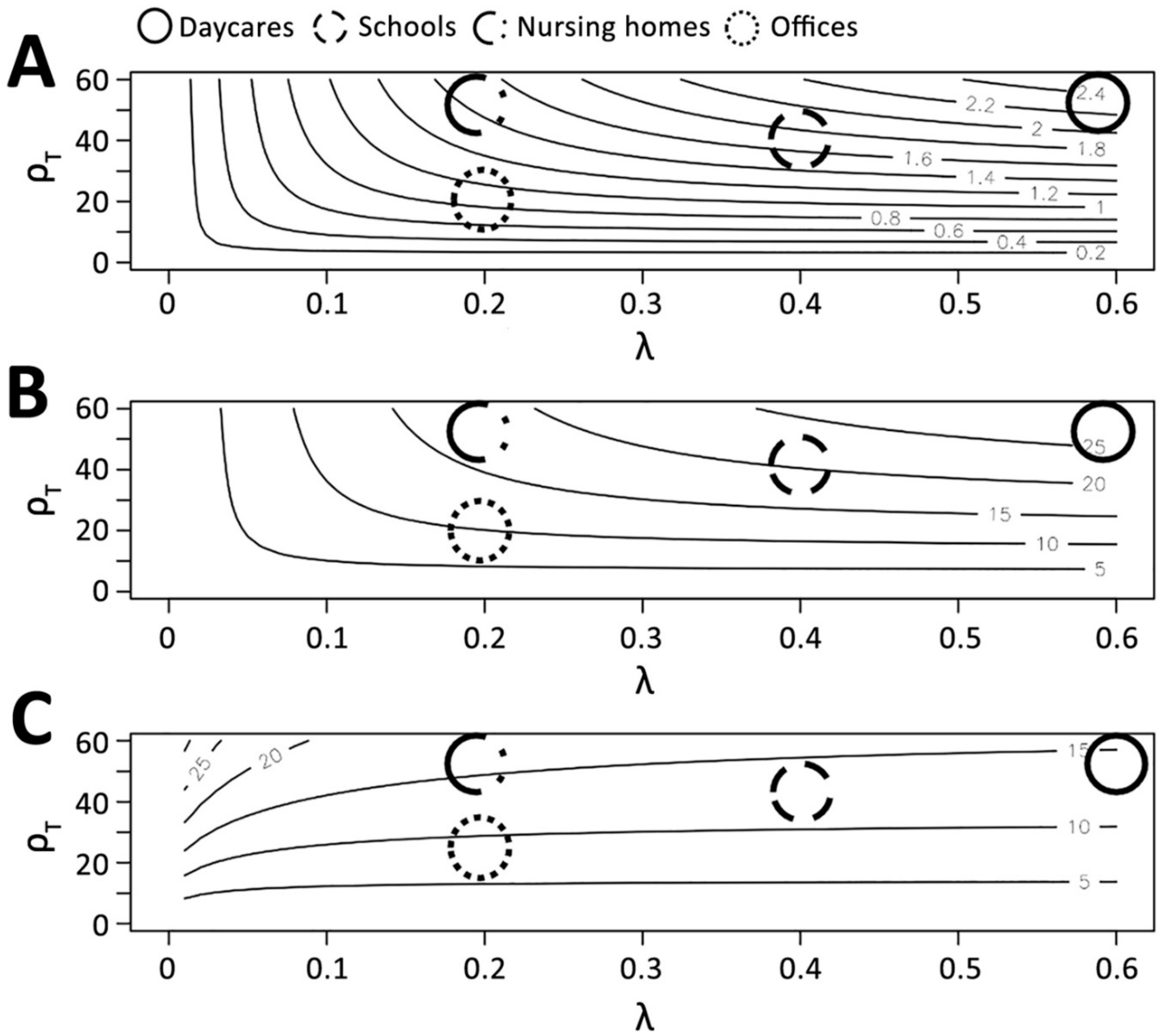

Appendix Figure 6. Predicted basic reproduction number for the fomite pathway for A) influenza, B) severe acute respiratory syndrome coronavirus 2, and C) rhinovirus without any interventions, by setting. Hourly fomite touching rates (ρ_T) and proportion of accessible surfaces (λ) are not known precisely, so larger circular symbols are used to reflect uncertainty, highlighting the plausible range. All 3 pathogens are shown using decay rates from stainless steel surfaces. Parameters used for each pathogen are shown in Appendix Figure 1.
